# Precision Oncology Guided by Genomic Profiling in Breast Cancer: Real-World Data from a Molecular Tumor Board

**DOI:** 10.3390/cancers17152435

**Published:** 2025-07-23

**Authors:** Tim Graf, Laura A. Boos, Tarun Mehra, Nicola Miglino, Bettina Sobottka, Jan H. Rüschoff, Luis Fábregas-Ibáñez, Martin Zoche, Heike Frauchiger-Heuer, Isabell Witzel, Alexander Ring, Andreas Wicki

**Affiliations:** 1 Department of Medical Oncology and Hematology, University Hospital Zurich, 8091 Zurich, Switzerland; 2 Faculty of Medicine, University of Zurich, 8006 Zurich, Switzerland; 3 Department of Pathology and Molecular Pathology, University Hospital Zurich, 8091 Zurich, Switzerland; 4 Department of Gynecology, University Hospital Zurich, 8091 Zurich, Switzerland

**Keywords:** precision oncology, next-generation sequencing, genomic profiling, breast cancer, molecular tumor board, matched targeted therapy, real-world evidence

## Abstract

Background/Objectives: Next-generation-sequencing-based genomic profiling (GP) of advanced breast cancer (BC) has been increasingly integrated into clinical practice. The growing number of biomarker-based therapies in BC increasingly complicates treatment decisions. As a result, molecular tumor boards (MTBs) have become pivotal. However, real-world data on the utility of MTBs in advanced BC remain limited. This study evaluates the translation of molecular findings in BC patients into MTB recommendations and examines their implementation and outcomes in real-world clinical practice. Methods: This retrospective, single-center study included 103 BC patients who received GP between January 2018 and December 2023. Patients were discussed at the weekly multidisciplinary MTB of our institution. Data retrieved included patient characteristics, GP results, and MTB recommendations, which were consecutively matched with treatment outcomes, namely the proportion of patients receiving an MTB treatment recommendation, proportion of patients receiving molecularly matched targeted therapy (MTT), and best treatment response. Results: The MTB reviewed 94 patients and provided 155 recommendations to 68 patients (72.3%), including systemic anti-cancer treatment (*n* = 123), clinical study participation (*n* = 4), genetic counseling (*n* = 12), and additional molecular testing (*n* = 16) recommendations. Treatment recommendations were provided to 63 patients (67%), of whom 38 (60.3%) received MTT. Of the 35 patients eligible for response assessment, 16 (45.7%) demonstrated clinical benefit: three achieved a complete response, six a partial response, and ten a stable disease > 6 months. Conclusions: GP and MTBs expand biomarker-matched treatment options to BC patients beyond the standard of care. Around half of the patients who receive MTT experience a clinical benefit. The standardization of procedures, the development of multi-biomarker-based prediction, and the enhancement in MTT delivery to patients are key challenges, which should be addressed in future initiatives.

## Simple Summary

Precision oncology involves tailoring cancer treatment to a tumor’s molecular profile. In patients with advanced breast cancer, who have exhausted standard-of-care treatments, tumor samples are increasingly tested for traditional predictive biomarkers (e.g., hormone receptors and HER2 amplifications). In this study, we performed genetic profiling in 103 breast cancer patients using an NGS assay covering 324 cancer-relevant genes. The results were discussed in our multidisciplinary molecular tumor board. More than two-thirds of patients were provided with a systemic anti-cancer treatment recommendation. Approximately 60% of patients with a treatment recommendation received a targeted therapy matched to the genetic profile, of whom around 45% benefitted clinically. This study contributes to the real-world evidence supporting the role of molecular tumor boards in providing personalized treatment to breast cancer patients with otherwise limited treatment options.

## 1. Introduction

Precision oncology, the concept of enhancing cancer treatment by tailoring therapies to a tumor’s molecular alterations, marked a paradigm shift in medical oncology [1]. Since the first sequencing of a human [2] and subsequently of cancer [3] genomes were completed in 2001 and 2006, respectively, next-generation sequencing (NGS) has become the foundation for precision oncology. Over the last decade, performing NGS has become much more rapid and cost-effective. This progress was highlighted by the UK’s 100,000 Genomes Project, which provided whole-genome sequencing information for 13,880 solid tumors covering 33 cancer types, including 2925 cases of breast cancer (BC) [4]. It revealed that in 49% of BCs, a clinically relevant mutation was present. These results are in line with the growing number of biomarker-based drug approvals in BC, which contributed to the improvement in response and survival in patients with metastatic BC [5].

While the therapy of patients with advanced BC was historically limited to chemo- and endocrine therapy with relatively poor outcomes, the therapeutic options now range from immune checkpoint inhibitors (ICIs) to poly ADP ribose polymerase (PARP), cyclin-dependent kinase (CDK) 4/6, mammalian target of rapamycin (mTOR), AKT, and phosphoinositide 3-kinase (PI3K) inhibitors. Among biomarker-driven therapies, the PI3K inhibitor alpelisib in combination with fulvestrant prolonged progression-free survival (PFS) in PIK3CA-mutated, hormone receptor (HR)-positive (+), and human epidermal growth factor receptor 2 (HER2)-negative (−) BC [6]. Also, PARP inhibitors, originally approved for HER2− metastatic BC in germline *BRCA-1/2* mutation carriers, are now being investigated in patients with homologous recombination repair (HRR)-deficient BC, showing promising results [7,8,9]. The response to ICIs has been associated with high tumor mutational burden (TMB) [10], which led to the US Food and Drug Administration’s (FDA) approval of pembrolizumab in TMB-high solid tumors [11]. Advancements are continuing with the FDA’s approval of the pan-AKT inhibitor capivasertib for *PIK3CA/AKT1/PTEN*-mutated HR+/HER2− BCs [12] and elacestrant for *ESR1*-mutated HR+/HER2− BCs [13].

Due to the progress in NGS and matched targeted therapies (MTTs) in recent years, NGS-based genomic profiling (GP) of advanced BC has seen growing integration into clinical practice [14]. Compared to smaller NGS hotspot panels, GP with large panels (>100 genes) yields more information on complex or rare biomarkers and includes genomic signatures. While this may lead to a broader range of treatment options, the clinical interpretation of vast molecular data with potentially multiple predictive markers and the application to clinical cancer care present a major challenge. To this end, molecular tumor boards (MTBs) have emerged as key players in translating molecular findings into clinical recommendations [15]. Several studies have demonstrated that multidisciplinary MTBs effectively guide MTTs and offer clinical utility in a wide range of solid tumors [16,17]. There is, however, limited real-world data available on the utility of MTBs in advanced BC.

We therefore decided to evaluate the translation of molecular findings into MTB recommendations in 103 BC patients who underwent GP with FoundationOne^®^CDx (F1^®^CDx) (Foundation Medicine Inc., Cambridge, MA, USA) from January 2018 to December 2023. Additionally, we report the implementation and outcomes of these recommendations in real-world clinical practice.

## 2. Materials and Methods

### 2.1. Study Population and Design

This retrospective, single-center study is part of a project continuously assessing MTB recommendations and corresponding patient outcomes. The cohort of the present study includes all patients with BC at the Comprehensive Cancer Center Zurich who received F1^®^CDx testing from January 2018 (the time of its implementation in the center) to December 2023. Patients who did not provide written general consent for research, or for whom no clinical record was available, were excluded. The retrospective data collection was conducted according to the guidelines of the Declaration of Helsinki and was approved by the local ethics committee (BASEC: 2021-01584).

The objective of this descriptive study was to evaluate (i) the genomic profile of patients with BC referred to the MTB; (ii) the recommendations provided by the MTB based on GP; (iii) the implementation of treatment recommendations by the treating physician; and (iv) the outcome and response to MTT. Outcome measures included the proportion of patients receiving an MTB (treatment) recommendation, the proportion of patients receiving MTT, their best response (stable disease (SD)/progressive disease (PD) in <6 months, SD for >6 months, partial response (PR), or complete response (CR)), PFS, PFS-ratio (comparison of PFS under MTT to PFS under the prior therapy, with a ratio ≥1.3 indicating clinical benefit for the individual patient as described by Von Hoff et al. [18]), and overall survival (OS).

### 2.2. Genomic Profiling

GP was conducted using F1^®^CDx. For this assay, DNA was extracted from routinely collected formalin-fixed, paraffin-embedded (FFPE) tumor tissue samples. A minimum of 20% tumor content was confirmed through histological review prior to DNA extraction.

DNA was extracted from tumor tissue using the Promega Maxwell RSC FFPE Plus DNA Kit, following the manufacturer’s protocol. Extracted DNA was quantified using a Qubit Fluorometer (Thermo Fisher Scientific, Waltham, MA, USA).

Library preparation was performed using the FoundationMedicine-targeted enrichment protocol to capture coding regions of interest. The preparation included the fragmentation of genomic DNA (200 bp insert size) followed by adapter ligation, and target enrichment by hybridization with custom-designed baits [19].

Next-generation sequencing was conducted on the Illumina^®^ (Illumina, San Diego, CA, USA) NovaSeq 6000 sequencing platform. Comprehensive, standardized deep sequencing was performed to high uniform depth (>500× median coverage with >99% of exons at coverage >100×). The individual analysis pipeline detects all classes of genomic alterations, including base substitutions, insertions, deletions, copy number alterations, and genomic rearrangements. The assay covers the exons of 324 genes and introns of 36 genes, which frequently show alterations in multiple solid tumors. A complete list of the covered genes is shown in Table S1. In addition, genomic signatures including microsatellite instability (MSI), TMB, and loss of heterozygosity (LOH) score are reported.

### 2.3. Molecular Tumor Board

The weekly MTB at our institution involves a multidisciplinary group of healthcare professionals. It comprises medical oncologists, pathologists, bioinformaticians, human geneticists, molecular biologists, and organ specialists (for instance, gynecologists). After case presentation, the MTB reviewed its clinical data, previous treatments, performance status, and genomic profile. The visualization of genomic profiles was supported by the Molecular Tumor Profiling pilot (MTPpilot) webservice developed by the University Hospital Zurich, which has been described previously [20]. Recommendations were issued accordingly, considering SOC treatments, regulatory labels, evidence from clinical trials and cohorts, drug availability, and ongoing clinical trials at the Comprehensive Cancer Center Zurich and other cancer centers. The discussion and recommendations were provided in a structured MTB report. All recommendations were advisory, with the final decision regarding diagnostic procedures and therapies being made by the treating physician and the patient.

### 2.4. Data Collection

Patient data were retrospectively curated, structured, and aggregated using a project-specific tool and stored in a relational database. Our tool facilitated the structured curation of clinical records extracted from the clinical information system KISIM (CISTEC AG, Zurich, Switzerland). Data curation was performed manually through electronic forms utilizing standardized terminologies and data models based on the Minimal Common Oncology Data Elements (mCODE) standard [21]. Additionally, all GP-related data were automatically aggregated from structured F1-reported results by the tool. The last follow-up was on the 8 July 2024.

Age, stage, and performance status were determined at the time of first GP. The Charlson Comorbidity Index [22] was calculated at the time of diagnosis. Histology was determined according to the latest classification of breast tumors of the World Health Organization [23]. Grading was performed by determining the Bloom–Richardson–Elston Score [24]. HER2 testing followed the American Society of Clinical Oncology/College of American Pathologists guidelines published in 2018 [25]. The immunohistochemistry assay was performed with an FDA-approved antibody (PATHWAY^®^ anti-HER2/neu 4B5, Ventana (Ventana Medical Systems Inc., Marana, AZ, USA)). Estrogen receptor (ER) and progesterone receptor (PR) assessment was performed by validated immunohistochemistry. The immunoreactivity of ≥10% of tumor cell nuclei was defined as ER/PR-positivity, given the limited evidence of endocrine therapy benefit below this threshold [26]. If several histological and immunohistochemistry assessments of the same cancer were available, the closest to GP was used.

High TMB was defined as TMB ≥ 10 mutations per megabase (Mut/Mb) since this threshold has been set by the FDA for the tumor-agnostic approval of pembrolizumab [11]. A high LOH score was defined as an LOH score ≥16 as proposed in patients with ovarian cancer in the ARIEL3 trial [27]. HRR deficiency was inferred by the MTB based on the detected genomic alterations and expert interpretation.

Recommendations by the MTB had to be explicitly mentioned in the MTB report to be considered in the analysis. Assessment of whether a drug was SOC was performed according to the most recent European Society for Medical Oncology (ESMO) guidelines for the treatment of patients with metastatic BC [28]. The implementation of an MTB treatment recommendation was defined as the administration of a recommended therapy by the treating physician, using a dosage and schedule consistent with clinical standards.

### 2.5. Response Assessment and Clinical Outcome

Response assessment was based on retrospective analysis of radiology reports in analogy to the Response Evaluation Criteria in Solid Tumors (RECIST), version 1.1 [29]. Response assessment was only performed for treatments which were actually administered. PFS was calculated from the date of initiation of MTT or previous therapy to the date of PD, death, or last follow-up. If patients were lost to follow-up or did not progress at last follow-up, they were censored. Overall survival (OS) was calculated from the date of initiation of MTT to the date of death or last follow-up. If a patient received more than one MTT, the date of initiation of the first therapy was used for OS calculation. Clinical benefit was defined as a PFS of ≥6 months and a best response of either CR, PR, or SD > 6 months. This definition was chosen due to methodological concerns associated with using a PFS-ratio ≥1.3 as a surrogate for clinical efficacy [30].

### 2.6. Statistical Analysis

Data was extracted from the project-specific database using Microsoft^®^ Excel for Mac (Microsoft, Redmond, WA, USA). Statistical analysis was performed using R for Mac (version 4.4.1 2024-06-14; R Foundation, Vienna, Austria). Continuous variables are reported as median (range), and categorical data as frequencies with percentages (*n* (%)). For univariate analyses, Fisher’s exact test was used.

## 3. Results

### 3.1. Study Population and Baseline Characteristics

**Figure 1:**
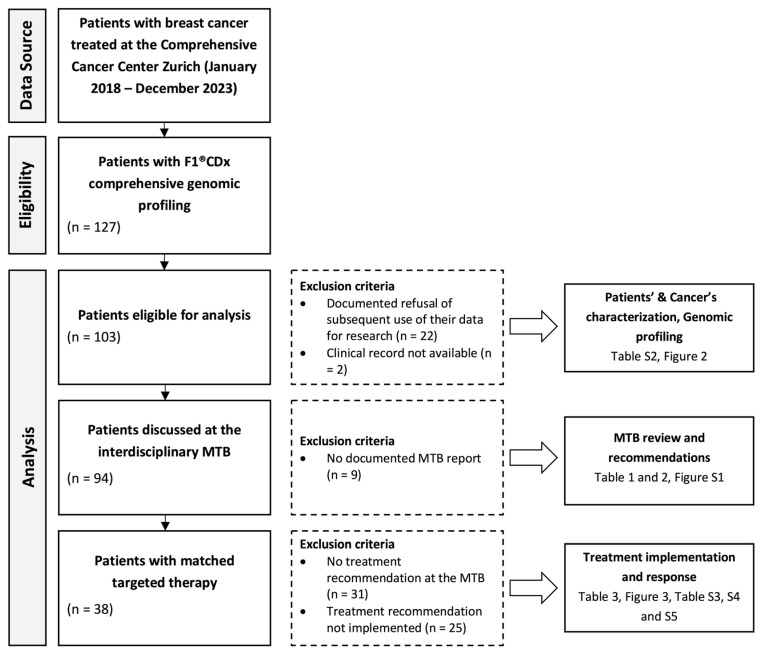
Study flow diagram. F1, FoundationOne; MTB, molecular tumor board.

Figure 1 details the Guidance for Reporting Oncology real-world evidence (GROW) diagram [31], demonstrating the flow of patients. From January 2018 to December 2023, 127 patients with BC received GP with F1^®^CDx.

A total of 103 patients were eligible for analysis after the exclusion of patients without documented general consent or clinical records. Overall, 102 patients (99%) were female. At the time of GP, the median age was 57 years (range, 36–86 years) and 95.1% of patients had stage IV BC. Most (72.8%) cancers were invasive breast carcinomas of no special type (NST). The most prevalent immunohistochemical BC subtype was HR+/HER2− (71.8%), followed by HR−/HER2− (22.3%), HR+/HER2+ (4.9%), and HR−/HER2+ (1%). Detailed clinicodemographic and cancer-specific characteristics are depicted in Table S2**.**

A total of 94 of the 103 patients with GP and informed consent were discussed at the weekly multidisciplinary MTB. In this group, 63 patients (67%) received a treatment recommendation, and 38 patients (40.4%) received MTT (Figure 1).

### 3.2. Genomic Profiling Results

**Figure 2:**
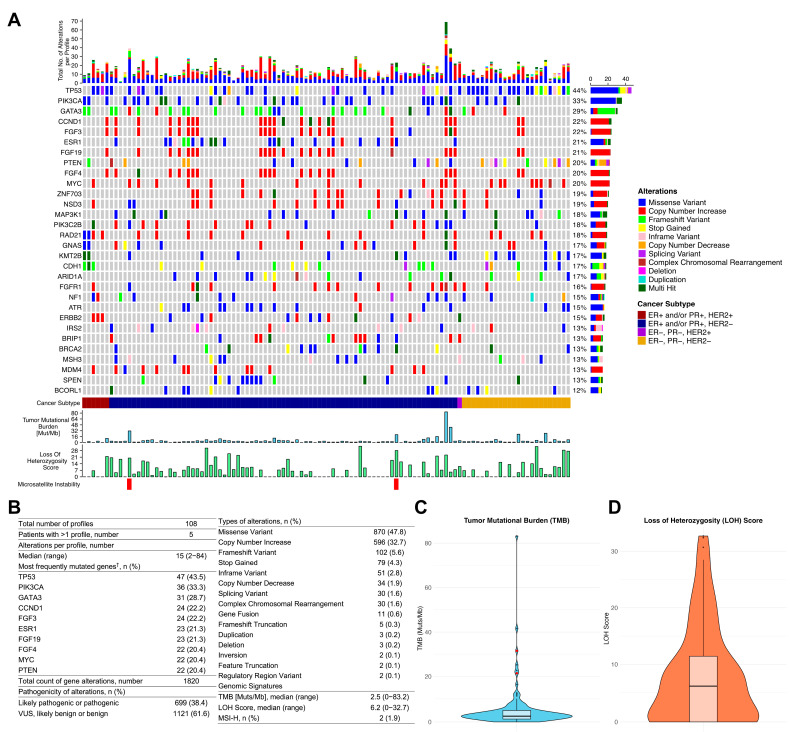
Results of genomic profiling (*n* = 108). (**A**) An oncoplot in which each vertical column represents one profile. On top, the total alterations per profile are shown. On the bottom, the cancer subtype, genomic signatures, and microsatellite status are depicted. Horizontally, the 30 most frequently mutated genes (from top to bottom) are shown. The percentage on the right represents the fraction of molecular profiles in which the respective gene is altered. The right boxplot displays the total number of alterations in each gene. (**B**) Summarized results of genomic profiling. (**C**) A violin plot illustrating tumor mutational burden. Outlier dots marked red represent profiles with high microsatellite instability. (**D**) A violin plot illustrating loss of heterozygosity score. F1, FoundationOne; VUS, variant of unknown significance; TMB, tumor mutational burden; LOH, loss of heterozygosity; MSI-H, high microsatellite instability. † refers to the count of molecular profiles in which the respective gene is altered.

GP was conducted by F1^®^CDx; the results are depicted in Figure 2. The total number of profiles was 108; five patients received two tests. The median number of molecular alterations per profile was 15 (range, 2–84). The most frequently altered genes were *TP53* (43.5%), *PIK3CA* (33.3%), and *GATA3* (28.7%). The most commonly observed *PIK3CA* mutations were the hotspot mutations E545K (*n* = 12), H1047R (*n* = 8), and E542K (*n* = 6). A total of 38.4% of the alterations were likely pathogenic or pathogenic. The median LOH score was 6.2 (range, 0–32.7). Two profiles (1.9%) showed high MSI. The median TMB was 2.5 Mut/Mb (range, 0–83.2 Mut/Mb). The patient with a TMB of 83.2 Mut/Mb also revealed multiple deleterious alterations in *BRCA1*, *ATM*, and *RAD51*, along with an alteration count of 84.

### 3.3. Molecular Tumor Board Review

Results of the MTB review are shown in Table 1. The MTB reviewed 94 patients and issued 155 recommendations to 68 patients (72.3%). The MTB provided the following types of recommendations: systemic anti-cancer treatment (*n* = 123), clinical study participation (*n* = 4), genetic counseling (*n* = 12), and additional molecular testing (*n* = 16) recommendations.

CUP, cancer of unknown primary; MTB, molecular tumor board; PI3K, phosphoinositide 3-kinase; PARP, poly (ADP-ribose) polymerase; FGFR, fibroblast growth factor receptor; mTOR, mammalian target of rapamycin; HER2, human epidermal growth factor receptor 2; CDK, cyclin-dependent kinase; MEK, Mitogen-activated protein kinase kinase; MAPK, Mitogen-activated protein kinase; SMO, Smoothened; SOC, standard of care; TMB, tumor mutational burden; LOH, loss of heterozygosity.

A total of 63 patients (67%) received a systemic anti-cancer treatment recommendation, either in anticipation of therapeutic drug efficacy (95.1%) or lack of efficacy (4.9%). The most frequently recommended drug classes were PI3K inhibitors (*n* = 34, 27.6%), endocrine therapy (*n* = 30, 24.4%), and PARP inhibitors (*n* = 13, 10.6%). The most common rationales for MTT recommendations were *PIK3CA* alterations (*n* = 51, 41.5%), high TMB (*n* = 13, 10.6%), and *ESR1* alterations (*n* = 11, 8.9%). Figure S1 presents all therapeutic drug efficacy recommendations along with the underlying genomic alterations.

Out of 123 treatment recommendations, 33 (26.8%) were non-SOC (Table 2). All non-SOC recommendations were off-label, according to Swissmedic, the national authorization and supervisory authority for drugs in Switzerland. Among non-SOC recommendations, ICIs were recommended most frequently (*n* = 10, 30.3%), followed by fibroblast growth factor receptor (FGFR) inhibitors (*n* = 9, 27.3%) and PARP inhibitors (*n* = 7, 21.2%). Recommendations for ICIs were based on high TMB. FGFR inhibitor recommendations were mainly based on amplifications in *FGFR1* (*n* = 4) and *FGFR2* (*n* = 1). In one patient, an *FGFR3* S249C missense variant led to a recommendation for FGFR inhibition. This alteration is infrequently observed in BC but is particularly common in bladder cancer [32]. PARP inhibitors were primarily recommended due to a high LOH score (*n* = 4) or HRR-deficiency (*n* = 1). In one patient with triple-negative BC (TNBC) harboring a *PIK3CA* Q526R missense variant, a PI3K inhibitor was recommended. Other infrequent non-SOC recommendations included mTOR inhibitors in TNBC and MEK, SMO, and MAPK inhibitors based on a *BRAF-GTF2I* gene fusion, a *PTCH1* M1V stop-gained variant, and a *MAPK1* E322K missense variant, respectively.

### 3.4. Implementation of Drug Recommendations

In total, there were 52 treatments in 38 patients. Thus, 60.3% of patients with a treatment recommendation were treated accordingly.

Table 3 provides an overview of the MTTs with anticipated therapeutic drug efficacy, of which there were 48 in 35 patients. Seven treatments (14.6%) were non-SOC. The median palliative treatment line was 2 (range, 1–11). The median treatment duration was 6 months (range, 0–49 months). In 18.8% of patients, the treatment was ongoing at last follow-up.

Prescribed drug classes for MTT included endocrine therapy, PI3K inhibitors, PARP inhibitors, HER2-targeting antibodies and tyrosine kinase inhibitors (TKI), chemotherapy, ICIs, mTOR inhibitors, FGFR inhibitors, and CDK4/6 inhibitors. Endocrine therapy (*n* = 16), PI3K inhibitors (*n* = 14), and PARP inhibitors (*n* = 4) were the most frequently administered. MTTs with therapeutic drug efficacy, grouped by drug class, are shown in Table S3.

### 3.5. Response and Outcome

**Figure 3:**
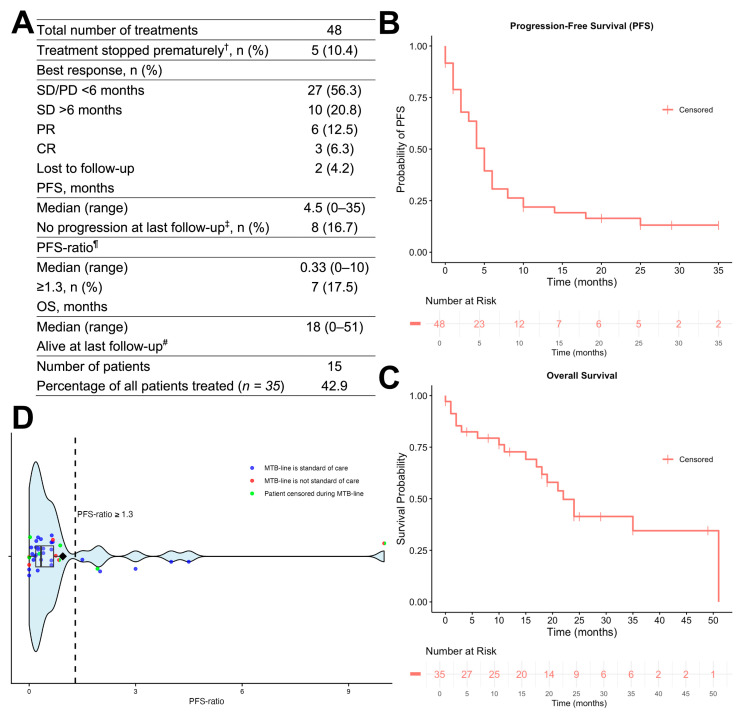
The response of patients treated with matched targeted therapy. (**A**) The table summarizes the outcome. (**B**) The probability of progression-free survival over time. (**C**) The probability of overall survival over time. (**D**) The raincloud plot depicts the distribution of the PFS-ratio, with individual data points scattered along the *y*-axis. The black box represents the median and interquartile range, and the diamond indicates the mean. A dashed vertical line marks the threshold for PFS-ratio ≥ 1.3. SD, stable disease; PD, progressive disease; PR, partial response; CR, complete response; PFS, progression-free survival; OS, overall survival. ^†^ Due to side effects or worsening of general condition. ^‡^ In two patients, the progression status is unknown. ^¶^ In eight cases, the ratio was not calculable—in seven cases, the MTB-line was a first line therapy, and in one case, the PFS of the previous line was zero months. Only patients with an available PFS-ratio were included in the reference population for the calculation of the fraction. ^#^ One patient was lost to follow-up.

Figure 3 depicts the response and outcome of patients with MTT. Treatments were grouped according to their best response in SD/PD in <6 months (*n* = 27, 56.3%), SD for >6 months (*n* = 10, 20.8%), PR (*n* = 6, 12.5%), and CR (*n* = 3, 6.3%). Two patients under treatment (4.2%) were lost to follow-up for response assessment. Death marked progression in six cases. The median PFS was 4.5 months (range, 0–35 months). The median OS was 18 months (range, 0–51 months). The 1-year OS rate was 72.7% (95% CI, 58.9–89.8%). At last follow-up, 16.7% of patients were progression-free and 42.9% were alive. Treatment outcome, grouped by drug class, is depicted in Table S4.

A total of 16 out of 35 patients (45.7%) or 19 out of 48 treatments (39.6%) showed clinical benefit, defined by a PFS of ≥6 months and a best response of CR, PR, or SD >6 months. In 7 out of 40 cases (17.5%) with calculable PFS-ratio, the PFS-ratio was ≥1.3. A total of 4 (25%) of the 16 patients with clinical benefit received a non-SOC treatment (Table 2). A high LOH score was significantly associated with clinical benefit (*p* = 0.009), whereas no significant associations were observed for other clinicopathological or molecular variables in univariate analysis (Table S5).

## 4. Discussion

With the increasing complexity associated with the use of large sequencing panels in clinical practice, MTBs are essential in interpreting their results and phrasing clinically useful recommendations. Current ESMO guidelines do not advise a broad multigene sequencing panel approach beyond the known biomarkers in BC [33]. Nevertheless, especially in cancer patients who have undergone SOC treatments, the trend goes towards GP and MTB discussion for treatment stratification, as supported by the latest guidelines for precision oncology by the Austrian, German, and Swiss Societies for hematology and medical oncology [34].

Our study provides one of the first and most comprehensive analyses of an MTB operating in real-world conditions with a comparably large cohort of BC patients. We used F1^®^CDx, a commercially available NGS-based test covering 324 genes for GP. In our study, 63 out of 94 patients (67%) discussed at the MTB received a treatment recommendation and 38 out of 63 patients (60.3%) received MTT. Reports with similar intention in BC patients have reported similar (58–66.3%) [35,36] or lower (20%) [37] recommendation rates, while some [38,39,40] have only reported the frequency of potentially actionable alterations instead of MTB recommendations. This distinction is important, as in real-world practice, not only potentially actionable alterations but also cancer subtype, stage, therapy line, drug availability, and performance status must be considered, since the real clinical benefit to individual patients is the relevant currency for such initiatives. Concerning treatment implementation, we found higher rates compared to similar studies in BC patients (28.9–58.9%) [35,36,37,40]. Causes for the non-implementation of MTB treatment recommendations were not included in our data collection, since this data is not routinely collected in clinical practice. However, previous work on this subject has identified several factors with varying frequencies, including reimbursement issues, ongoing response to the previous treatment line, decision for best-supportive care, use of alternative treatments, clinical deterioration, and death [41,42]. This highlights the importance of the early initiation and quick processing of genomic analyses and MTB discussions, as the patients undergoing these tests may experience relatively rapid disease progression.

In our cohort, 16 out of 35 patients (45.7%) or 19 out of 48 treatments (39.6%) eligible for response assessment showed clinical benefit. Previous research has shown comparable responses to MTT in BC patients (29–46.2%), although the definition of clinical benefit is not consistent across these studies [35,41,43,44]. Few randomized trials assess precision oncology outcomes in BC. The SAFIR02-BREAST study [45], a prospective randomized trial, has compared MTTs to SOC treatments in patients with metastatic BC. It has reported a PFS benefit for treatments matched to level I/II alterations according to the ESMO Scale for Clinical Actionability of Molecular Targets (ESCAT) [46], but not beyond. Frameworks such as the ESCAT and the Memorial Sloan Kettering Cancer Center Precision Oncology Knowledge Base (OncoKB) [47] contribute to the standardization of alteration interpretation across MTBs in different institutions. Similarly, the ESMO Precision Medicine Working Group recommendations [48] should facilitate the identification of potential germline variants in tumor-only NGS, which is also a key task of the MTB.

Seven patients received a non-SOC treatment. These were implemented less frequently than SOC recommendations but may have yielded greater clinical benefit. Four patients showed clinical benefit, while the other three died shortly after treatment initiation due to treatment-unrelated causes. While SOC recommendations are often guided by well-established alterations such as mutations in *PIK3CA*, *ESR1*, and *BRCA1/2*, non-SOC recommendations target less commonly addressed pathways and are usually supported only by anecdotal evidence. This is reflected by the greater challenge in implementing such recommendations, which typically requires off-label use of a drug approved for a different cancer entity. One example is FGFR inhibitors, which were recommended in our study primarily due to amplifications in *FGF* receptors or ligands. Their role in breast cancer is yet unclear, and a clinical benefit has only been proven in other entities such as cholangiocarcinoma and urothelial carcinoma, but not for gene amplifications [49]. In our cohort, only one patient with HR−/HER2− BC was treated with an FGFR inhibitor, with poor response. In the case of ICIs, which were recommended as non-SOC in TMB-high BC, there are regional regulatory differences. The FDA approved pembrolizumab for TMB-high solid tumors, unlike in Europe, where no ICI is approved for this indication. Of note, the KEYNOTE-158 study [10], which formed the basis for the FDA’s approval, did not include BC patients, and evidence supporting the effectiveness of pembrolizumab monotherapy in TMB-high BC is limited to the nonrandomized phase 2 TAPUR study and expert consensus [50,51]. Notably, in our study, one patient with HR+/HER2− BC and a TMB of 12.6 Mut/Mb received pembrolizumab in the third line, achieving an ongoing PFS of 20 months. The RUBY trial established a link between high LOH, indicative of HRR-deficiency, and response to rucaparib in endometrial cancer [7]. However, this connection remains speculative for breast cancer. One patient in our cohort with HR+/HER2− BC and high LOH achieved CR on olaparib in the third line, remaining progression-free at last follow-up. Another patient with HR+/HER2− BC, who received talazoparib and olaparib consecutively due to HRR-deficiency, achieved PR and an 8-month PFS. Recently, monotherapy with olaparib has been investigated in HR−/HER2− HRR-deficient BC, with three of six patients demonstrating a PR [52]. Additionally, preclinical data have demonstrated that PARP inhibitors may increase TMB, potentially predicting therapeutic response to ICIs [53]. As a result, the combination of PARP inhibitors with ICIs is being explored in various tumor types, with promising preliminary results [54]. Presently, however, no consensus exists on defining HRR-deficiency, despite its importance in predicting PARP inhibitor sensitivity [55]. Hence, the MTB remains crucial for identifying HRR-deficient cancer phenotypes and enabling such non-SOC treatments.

To our knowledge, previous studies with similar intention have not focused on non-SOC recommendations or treatments, making comparisons difficult. However, our data suggest that especially in patients beyond the SOC, GP and MTBs allow for meaningful clinical benefits, although these findings require prospective validation in larger cohorts. It must also be noted that this single biomarker approach does not adequately represent the complexity of tumor biology. This limitation is reflected by a considerable proportion of patients who fail to respond to genotype-matched therapy. Recent technological advances enabling the high-resolution analysis of molecular structures and functional phenotypes at both the cellular and subcellular levels offer the potential for a more comprehensive characterization of tumor heterogeneity and environment [56]. Innovative clinical trials are currently underway to prospectively evaluate predictive biomarkers beyond genetic alterations [57,58]. The introduction of multi-biomarker-based predictive models holds the potential to redefine the precision oncology paradigm.

Our study has several limitations. The descriptive, retrospective, single-center design may introduce significant biases. In particular, selection bias may have occurred due to the exclusion of patients without documented consent, clinical records, or MTB reports. Additionally, our patient population showed significant heterogeneity in terms of NGS timing, number and type of previous therapy lines, and follow-up intervals. Consequently, the creation of a valid propensity-matched control group was not feasible, which limits the generalizability of our results. Lastly, the sample size of our study, particularly of the group receiving MTT, was small, which limits the statistical significance of any conclusions regarding response and outcome to MTT. However, the heterogeneity and size of our cohort reflect the real-world conditions in which MTBs operate. In this respect, structured data collection for data exchange and cross-institutional collaboration should be pursued to improve patient care and potentially enhance patient outcomes. Moreover, it could contribute to the standardization of procedures related to the choice of sequencing panels, alteration interpretation, and delivering treatment to patients.

## 5. Conclusions

In conclusion, we found that MTBs can provide additional treatment options to patients with otherwise limited further treatment lines, leading to clinical benefits in around half of patients. The standardization of procedures, the introduction of multi-biomarker-based predictive models, and the enhancement in MTT delivery to patients are key challenges, which should be addressed in future initiatives.

## Figures and Tables

**Table 1:** Molecular tumor board reviews and recommendations.

MTB reviews, number	104
Molecular profile analysis, *n* (%)	
Molecular comparison conducted	2 (1.9)
CUP characterization conducted	0 (0)
MTB review with recommendation, *n* (%)	72 (69.2)
Total count of MTB recommendations, number	155
Type of MTB recommendation, *n* (%)	
Treatment	123 (79.4)
Clinical study	4 (2.6)
Genetic counseling	12 (7.7)
Additional testing	16 (10.3)
Clinical study recommendations (*n* = 4)	
Study phase, *n* (%)	
Phase 1	1 (25)
Phase 2	3 (75)
Underlying gene alteration, *n* (%)	
*AKT1*	2 (50)
*BRAF-GTF2I*	1 (25)
*FANCA*	1 (25)
Treatment recommendations (*n* = 123)	
Expected drug action, *n* (%)	
Therapeutic drug effect	117 (95.1)
Lack of drug effect	6 (4.9)
Drug class, *n* (%)	
PI3K inhibitor	34 (27.6)
Endocrine therapy	30 (24.4)
PARP inhibitor	13 (10.6)
Immune checkpoint inhibitor	11 (8.9)
FGFR inhibitor	9 (7.3)
Chemotherapeutic agent	7 (5.7)
mTOR inhibitor	7 (5.7)
HER2-targeting antibody	5 (4.1)
CDK4/6 inhibitor	2 (1.6)
HER2-targeting TKI	2 (1.6)
MEK inhibitor	1 (0.8)
MAPK inhibitor	1 (0.8)
SMO inhibitor	1 (0.8)
Adherence to clinical practice, *n* (%)	
Non-SOC	33 (26.8)
Most frequent underlying alteration, *n* (%)	
*PIK3CA*	51 (41.5)
High TMB	12 (9.8)
*ESR1*	11 (8.9)
*ERBB2*	8 (6.5)
High LOH score	6 (4.9)
*BRCA2*	5 (4.1)
*AKT1*	4 (3.3)
*FGFR1*	4 (3.3)
*BRCA1-NSRP1*	2 (1.6)

**Table 2:** Non-standard-of-care systemic anti-cancer treatment recommendations.

Cancer Subtype	Drug Class	Altered Gene	Type of Alteration	Protein Sequence Variation	Specific Drug	Palliative Line	PFS (m)	PFS-Ratio	OS (m)	Best Response
HR−/HER2−	FGFR Inhibitor	*FGFR1*	Copy Number Increase	—	Erdafitinib	8	1	— ^#^	2	SD/PD < 6 m
HR+/HER2−	FGFR Inhibitor	*FGFR1*	Copy Number Increase	—	—	—	—	—	—	—
HR+/HER2−	FGFR Inhibitor	*FGFR1*	Copy Number Increase	—	—	—	—	—	—	—
HR+/HER2−	FGFR Inhibitor	*FGFR1*	Copy Number Increase	—	—	—	—	—	—	—
HR+/HER2−	FGFR Inhibitor	*FGFR2*	Copy Number Increase	—	—	—	—	—	—	—
HR+/HER2−	FGFR Inhibitor	*FGFR3*	Missense Variant	p.(Ser249Cys)	—	—	—	—	—	—
HR+/HER2−	FGFR Inhibitor	*FGF3*	Copy Number Increase	—	—	—	—	—	—	—
HR+/HER2−	FGFR Inhibitor	*FGF4*	Copy Number Increase	—	—	—	—	—	—	—
HR+/HER2−	FGFR Inhibitor	*FGF19*	Copy Number Increase	—	—	—	—	—	—	—
HR+/HER2−	HER2-targeting TKI	*ERBB2*	Missense Variant	p.(Asp769Tyr)	—	—	—	—	—	—
HR−/HER2−	ICI	—	High TMB ^‡^	—	—	—	—	—	—	—
HR+/HER2−	ICI	—	High TMB	—	—	—	—	—	—	—
HR−/HER2−	ICI	—	«High» TMB (7.9 Mut/Mb)	—	—	—	—	—	—	—
HR+/HER2−	ICI	—	High TMB	—	—	—	—	—	—	—
HR+/HER2−	ICI	—	High TMB	—	—	—	—	—	—	—
HR+/HER2−	ICI	—	High TMB	—	—	—	—	—	—	—
HR+/HER2−	ICI	—	High TMB	—	Pembrolizumab	3	20 *	10	20 *	SD > 6 m
HR+/HER2−	ICI	—	High TMB	—	Ipilimumab/Nivolumab	5	0	0	0	SD/PD < 6 m ^†^
HR+/HER2−	ICI	—	High TMB	—	—	—	—	—	—	—
HR−/HER2−	ICI	—	High TMB	—	Atezolizumab	3	0	0	0	SD/PD < 6 m ^†^
HR−/HER2−	MAPK Inhibitor	*MAPK1*	Missense Variant	p.(Glu322Lys)	—	—	—	—	—	—
HR+/HER2−	MEK Inhibitor	*BRAF-GTF2I*	Gene Fusion	—	—	—	—	—	—	—
HR−/HER2−	mTOR Inhibitor	*PIK3R1*	Missense Variant	p.(Asn564Asp)	—	—	—	—	—	—
HR−/HER2−	mTOR Inhibitor	*PTEN*	Frameshift Variant	p.(Val222fs*1)	—	—	—	—	—	—
HR+/HER2−	PARP Inhibitor	—	High LOH Score ^‡^	—	—	—	—	—	—	—
HR+/HER2−	PARP Inhibitor	—	High LOH Score	—	—	—	—	—	—	—
HR+/HER2−	PARP Inhibitor	—	High LOH Score	—	Olaparib	3	21 *	0.84	21 *	CR
HR+/HER2−	PARP Inhibitor	—	High LOH Score	—	—	—	—	—	—	—
HR+/HER2−	PARP Inhibitor	*BRIP1*	Frameshift Variant	p.(Lys998fs*60)	—	—	—	—	—	—
HR+/HER2−	PARP Inhibitor	*CCND1*	Copy Number Increase	—	—	—	—	—	—	—
HR+/HER2−	PARP Inhibitor	—	HRR-deficiency	—	Talazo- and olaparib (consecutively)	3	8	0.67	18	PR
HR−/HER2−	PI3K Inhibitor	*PIK3CA*	Missense Variant	p.(Gln546Arg)	Alpelisib	2	6	0.75	12 *	PR
HR+/HER2−	SMO Inhibitor	*PTCH1*	Stop Gained	p.(Met1Val)	—	—	—	—	—	—
Summary, median (range)						3 (2–8)	6 (0–21)	0.71 (0–10)	12 (0–21)	—

PFS, progression-free survival; m, months; OS, overall survival; HR, hormone receptor; HER2, human epidermal growth factor receptor 2; FGFR, fibroblast growth factor receptor; SD, stable disease; PD, progressive disease; FGF, fibroblast growth factor; ICI, immune checkpoint inhibitor; TMB, tumor mutational burden; MAPK, Mitogen-activated protein kinase; MEK, Mitogen-activated protein kinase kinase; GTF2I, general transcription factor IIi; mTOR, mammalian target of rapamycin; PIK3R1, phosphoinositide-3-kinase regulatory subunit 1; PTEN, phosphatase and tensin homolog; PARP, poly (ADP-ribose) polymerase; LOH, loss of heterozygosity; CR, complete remission; PR, partial remission; HRR, homologous recombination repair; PI3K, phosphoinositide 3-kinase; PIK3CA, phosphatidylinositol-4,5-bisphosphate 3-kinase, catalytic subunit alpha; SMO, Smoothened; PTCH1, protein patched homolog 1. ^#^ The PFS-ratio was not calculable, since the PFS of the previous line was 0 months; ^‡^ high TMB was defined as TMB ≥ 10 Mut/Mb and high LOH score was defined as LOH score ≥ 16. * PFS/OS ongoing. ^†^ The patient died shortly after first the dose of ICI.

**Table 3:** Matched targeted therapies with anticipated therapeutic drug efficacy.

Matched Targeted Therapy	
Number of treatments	48
Percentage of all therapeutic drug effect recommendations (*n* = 117)	41
Number of patients	35
Percentage of all patients receiving a therapeutic drug effect recommendation (*n* = 59)	59.3
Adherence to clinical practice	
Non-SOC treatment recommendation followed, number	7
Percentage of all non-SOC treatment recommendations (*n* = 33)	21.2
Palliative treatment line	
Median (range)	2 (1–11)
Line 1, *n* (%)	7 (17.1)
Line 2, *n* (%)	18 (43.9)
Line 3, *n* (%)	13 (31.7)
Line 4, *n* (%)	5 (12.2)
Line 5, *n* (%)	3 (7.3)
Line 8, *n* (%)	1 (2.4)
Line 11, *n* (%)	1 (2.4)
Treatment duration, months	
Median (range)	6 (0–49)
Treatment ongoing ^†^, *n* (%)	9 (18.8)

SOC, standard of care; MTB, molecular tumor board. ^†^ Last follow-up on the 8 July 2024.

## Data Availability

The data are contained within the article or Supplementary Material. Additional data can be provided upon reasonable request.

## Supplementary Materials

The following supporting information can be downloaded at https://www.mdpi.com/article/10.3390/cancers17152435/s1. Table S1: The FoundationOne^®^CDx assay analyzes 324 genes, consisting of 309 genes with full exonic coverage and 15 genes with partial non-exonic coverage, marked with an *. Additionally, 75 genes, highlighted in bold, are captured with enhanced sensitivity and have full exonic coverage, unless specified otherwise. Figure S1: The Sankey diagram displays the recommended drug class by the molecular tumor board on the right. It connects each recommendation to a genomic feature on the left, whose alteration led to the recommendation. Only drug recommendations with a therapeutic drug effect are shown (*n* = 117). Endocrine therapy based on a PIK3CA alteration was always recommended in combination with a PI3K-Inhibitor. Table S2: Clinicodemographic and cancer-specific characteristics of breast cancer patients. Table S3: Matched targeted therapies with a therapeutic drug effect, grouped by drug class. Table S4: Outcomes of patients treated according to recommendations, grouped by drug class. Table S5: Univariate analysis of clinical benefit associated with clinicopathological or molecular variables.
