# Supplementary material for: Precision Oncology Guided by Genomic Profiling in Breast Cancer: Real-World Data from a Molecular Tumor Board
Source: Cancers (Basel). 2025 Jul 23;17(15):2435. doi: 10.3390/cancers17152435 (PMC12346032; doi:10.3390/cancers17152435)
Supplement: Supplementary file 1 [file cancers-17-02435-s001.zip › cancers-3659047-supplementary.pdf]

Table S1: The FoundationOne®CDx assay analyzes 324 genes, consisting of 309 genes with full exonic coverage and 15 genes with partial non-exonic coverage, marked with an *. Additionally, 75 genes, highlighted in bold, are captured with enhanced sensitivity and have full exonic coverage, unless specified otherwise.

ABL1 [Exons 4-9]	*CASP8*	***DDR2 [Exons 5,17,18]***	*FGFR4*	*KDR*	***MYD88 [Exon 4]***	*PPP2R2A*	***SMO***
ACVR1B	*CBFB*	*DIS3*	*FH*	*KEAP1*	*NBN*	*PRDM1*	*SNCAIP*
AKT1 [Exon 3]	*CBL*	*DNMT3A*	*FLCN*	*KEL*	***NF1***	*PRKAR1A*	*SOCS1*
AKT2	***CCND1***	*DOT1L*	*FLT1*	***KIT [Exons 8,9,11,12,13,17]***	*NF2*	*PRKCI*	*SOX2*
AKT3	*CCND2*	*EED*	***FLT3 [Exons 14,15,20]***	*KLHL6*	*NFE2L2*	*PTCH1*	*SOX9*
ALK [Exons 20-29, Introns 18,19]	*CCND3*	***EGFR [Introns*** *7,15,**24-27]***	***FOXL2***	*KMT2A (MLL)*	*NFKBIA*	***PTEN***	*SPEN*
ALOX12B	*CCNE1*	*EP300*	*FUBP1*	*KMT2D (MLL2)*	*NKX2-1*	***PTPN11***	*SPOP*
AMER1	*CD22*	*EPHA3*	*GABRA6*	***KRAS***	*NOTCH1*	*PTPRO*	*SRC*
APC	*CD70*	*EPHB1*	*GATA3*	*LTK*	*NOTCH2 [Intron 26]*	*QKI*	*STAG2*
AR	*CD74* {Introns 8-6}*	*EPHB4*	*GATA4*	*LYN*	*NOTCH3*	*RAC1*	*STAT3*
ARAF [Exons 4,5,7,11,13,15,16]	*CD79A*	***ERBB2***	*GATA6*	*VAF*	***NPM1 [Exons 4-6,8,10]***	*RAD21*	***STK11***
ARFRP1	*CD79B*	***ERBB3 [Exons 3,6,7,8,10,12,20,21,23,24,25]***	*GID4 (C17orf39)*	***MAP2K1 [Exons 2,3]***	***NRAS [Exons 2,3]***	*RAD51*	*SUFU*
ARID1A	***CD274***	*ERBB4*	***GNA11 [Exons 4,5]***	***MAP2K2 [Exons 2-4,6,7]***	*NT5C2*	*RAD51B*	*SYK*
ASXL1	*CDC73*	*ERCC4*	*GNA13*	*MAP2K4*	***NTRK1 [Exons 14,15, Introns 8-11]***	*RAD51C*	*TBX3*
ATM	***CDH1***	*ERG*	***GNAQ [Exons 4,5]***	*MAP3K1*	*NTRK2 [Intron 12]*	*RAD51D*	*TEK*
ATR	***CDK12***	***ERRFI1***	***GNAS [Exons 1,8]***	*MAP3K13*	***NTRK3 [Exons 16,17]***	*RAD52*	*TET2*
ATRX	***CDK4***	***ESR1 [Exons 4-8]***	*GRM3*	*MAPK1*	*NUTM1* {Intron 1}*	*RAD54L*	*TERC* {ncRNA}*
AURKA	***CDK6***	*ETV4* {Intron 8}*	*GSK3B*	*MCL1*	*P2RY8*	***RAF1 [Exons 3,4,6,7,10,14,15,17]***	***TERT* {Promoter}***
AURKB	*CDK8*	*ETV5* {Introns 6, 7}*	*H3F3A*	***MDM2***	***PALB2***	*RARA*	*TGFBR2*
AXIN1	*CDKN1A*	***ETV6* {Introns 5,6}***	*HDAC1*	*MDM4*	*PARK2*	***RB1***	*TIPARP*
AXL	*CDKN1B*	*EWSR1* {Introns 7, 13}*	*HGF*	*MED12*	*PARP1*	*RBM10*	*TMPRSS2* {Introns 1-3}*
BAP1	***CDKN2A***	***EZH2 [Exons 4,16,17,18]***	*HNF1A*	*MEF2B*	*PARP2*	*REL*	*TNFAIP3*
BARD1	*CDKN2B*	*EZR* {Introns 9-11}*	***HRAS [Exons 2,3]***	*MEN1*	*PARP3*	***RET [Exons 11,13-16, Introns 9,10,11]***	*TNFRSF14*
BCL2	*CDKN2C*	*FAM46C*	*HSD3B1*	*MERTK*	*PAX5*	*RICTOR*	***TP53***
BCL2L1	*CEBPA*	*FANCA*	*ID3*	***MET***	*PBRM1*	*RNF43*	*TSC1*

BCL2L2	*CHEK1*	*FANCC*	***IDH1 [Exon 4]***	*MITF*	*PDCD1*	***ROS1 [Exons 31,36-38,40, Introns 31-35]***	*TSC2*
BCL6	***CHEK2***	*FANCG*	***IDH2 [Exon 4]***	*MKNK1*	***PDCD1LG2***	*RPTOR*	*TYRO3*
BCOR	*CIC*	*FANCL*	*IGF1R*	*MLH1*	***PDGFRA [Exons 12,18]***	*RSPO2* {Intron 1}*	*U2AF1*
BCORL1	*CREBBP*	*FAS*	*IKBKE*	***MPL [Exon 10]***	***PDGFRB [Exons 12-21,23]***	*SDC4* {Intron 2}*	***VEGFA***
BCR {Introns 8, 13, 14}*	***CRKL***	*FBXW7*	*IKZF1*	*MRE11A*	*PDK1*	*SDHA*	*VHL*
BRAF [Exons 11-18]	*CSF1R*	*FGF10*	*INPP4B*	*MSH2*	*PIK3C2B*	*SDHB*	*WHSC1*
BRCA1	*CSF3R*	*FGF12*	*IRF2*	*MSH3*	*PIK3C2G*	*SDHC*	*WHSC1L1*
BRCA2	*CTCF*	*FGF14*	*IRF4*	*MSH6*	***PIK3CA [Exons 2,3,5-8,10,14,19,21 (Coding Exons 1, 2, 4-7, 9, 13,18,20)]***	*SDHD*	*WT1*
BRD4	*CTNNA1*	*FGF19*	*IRS2*	*MST1R*	*PIK3CB*	*SETD2*	*XPO1*
BRIP1	***CTNNB1 [Exon 3]***	*FGF23*	*JAK1*	*MTAP*	*PIK3R1*	*SF3B1*	*XRCC2*
BTG1	*CUL3*	*FGF3*	***JAK2 [Exons 14]***	***MTOR [Exons 19,30,39,40,43-45,47,48,53,56]***	*PIM1*	*SGK1*	*ZNF217*
BTG2	*CUL4A*	*FGF4*	***JAK3 [Exons 5,11,12,13,15,16]***	*MUTYH*	*PMS2*	*SLC34A2* {Intron 4}*	*ZNF703*
BTK [Exons 2,15]	*CXCR4*	*FGF6*	*JUN*	*MYB* {Intron 14}*	*POLD1*	*SMAD2*	
C11orf30	*CYP17A1*	***FGFR1***	*KDM5A*	***MYC***	*POLE*	*SMAD4*	
CALR	*DAXX*	***FGFR2 [Intron 17]***	*KDM5C*	*MYCL*	*PPARG*	*SMARCA4*	
CARD11	*DDR1*	***FGFR3 [Exons 7, 9 (alternative designation exon 10), 14, 18, Intron 17]***	*KDM6A*	***MYCN***	*PPP2R1A*	*SMARCB1*	

Table S2. Clinicodemographic and cancer-specific characteristics of breast cancer patients.

**Sex, n (%)**	
Female	102 (99)
Male	1 (1)
**Age at CGP, years**	
Median (range)	57 (34-86)
**Stage at CGP, n (%)**	
Stage IV	98 (95.1)
**Charlson Comorbidity Index, n (%)**	
<8	55 (53.4)
≥8	48 (46.6)
**ECOG PS Score†, n (%)**	
0	51 (49.5)
1	33 (32)
2	14 (13.6)
3	1 (1)
4	1 (1)
Missing data	3 (2.9)
**Patients with documented risk factor, n (%)**	
Germline mutation in BRCA1/2	2 (1.9)
Breast cancer in ≥1 first-degree relative	16 (15.5)
Smoking history of ≥10 py	21 (20.4)
Alcohol consumption ≥4 days per week	6 (4.9)
**Alive at last follow-up§, n (%)**	41 (41.4)
**Histology, n (%)**	
Invasive breast carcinoma of NST	75 (72.8)
Invasive lobular carcinoma	17 (16.5)
Other	11 (10.7)
**B.R.E. Score, n (%)**	
3-5 (= G1)	4 (3.9)
6-7 (= G2)	39 (37.9)
8-9 (= G3)	46 (44.7)
Missing data	14 (13.6)
**Subtype, n (%)**	
ER+ and/or PR+, HER2+	5 (4.9)
Ki-67‡ (%), median (range)	5 (5-5)
ER+ and/or PR+, HER2-	74 (71.8)
Ki-67‡ (%), median (range)	20 (1-80)
ER-, PR-, HER2+	1 (1)
Ki-67 (%)	55
ER-, PR-, HER2-	23 (22.3)
Ki-67 (%), median (range)	60 (10-95)

CGP, comprehensive genomic profiling; ECOG PS, Eastern Cooperative Oncology Group Performance Status; BRCA, Breast Cancer Gene; py, pack years; NST, no special type; B.R.E., Bloom-Richardson-Elston; ER, estrogen receptor; PR, progesterone receptor; HER2, human epidermal growth factor receptor 2; † The ECOG PS Score closest to CGP was used. § Last follow-up on the 8th of July 2024. Four patients were lost to follow-up. ‡ Information of Ki-67 expression is missing in 2 and 7 patients, respectively.

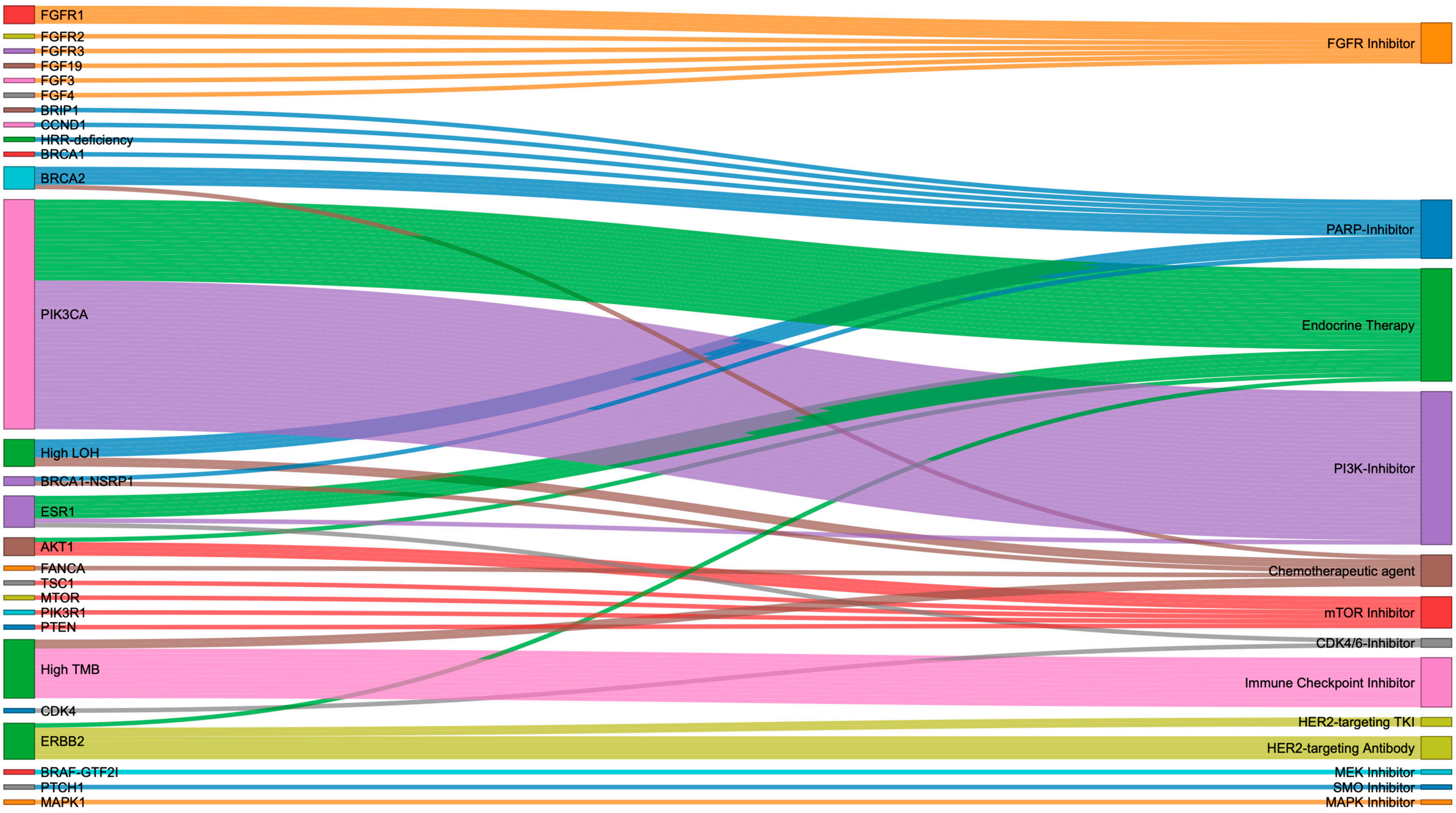

Figure S1: The Sankey diagram displays the recommended drug class by the Molecular Tumor Board on the right. It connects each recommendation to a genomic feature on the left, which or whose alteration led to the recommendation. Only drug recommendations with therapeutic drug effect are shown (n = 117). Endocrine therapy based on a PIK3CA alteration was always recommended in combination with a PI3K-Inhibitor.

Table S3: Matched targeted therapies with therapeutic drug effect, grouped by drug class.

Drug class:	PI3K Inhibitor	Endocrine therapy	PARP Inhibitor	CDK4/6 Inhibitor	HER2-targeting Antibody	HER2-targeting TKI	Chemo-therapy	Immune Checkpoint Inhibitor	mTOR Inhibitor	FGFR Inhibitor
**Matched targeted therapy**										
Number	14	16	4	1	3	1	3	3	2	1
Percentage of all recommendations of respective drug class	41.2	53.3	30.8	50	60	50	42.9	27.3	40	25
**Treatment commenced before the date of MTB review, n (%)**	1 (7.1)	5 (31.3)	0 (0)	0 (0)	3 (100)	0 (0)	0 (0)	0 (0)	0 (0)	0 (0)
**Adherence to clinical practice and regulatory label, n (%)**										
Non-SOC	1 (7.1)	0 (0)	2 (50)	0 (0)	0 (0)	0 (0)	0 (0)	0 (0)	0 (0)	1 (100)
Off-label	1 (7.1)	0 (0)	2 (50)	0 (0)	0 (0)	0 (0)	0 (0)	3 (100)	0 (0)	1 (100)
**Palliative treatment line**										
Median (range)	2.5 (2-4)	2 (1-4)	2.5 (2-3)	—	1 (1-5)	—	3 (2-3)	3 (3-5)	8 (5-11)	—
Line 1, n (%)	0 (0)	4 (25)	0 (0)	1 (100)	2 (66.7)	0 (0)	0 (0)	0 (0)	0 (0)	0 (0)
Line 2, n (%)	7 (50)	7 (43.8)	2 (50)	0 (0)	0 (0)	1 (100)	1 (33.3)	0 (0)	0 (0)	0 (0)
Line 3, n (%)	4 (28.6)	3 (18.8)	2 (50)	0 (0)	0 (0)	0 (0)	2 (66.7)	2 (66.7)	0 (0)	0 (0)
Line 4, n (%)	3 (21.4)	2 (12.5)	0 (0)	0 (0)	0 (0)	0 (0)	0 (0)	0 (0)	0 (0)	0 (0)
Line 5, n (%)	0 (0)	0 (0)	0 (0)	0 (0)	1 (33.3)	0 (0)	0 (0)	1 (33.3)	1 (50)	0 (0)
Line 8, n (%)	0 (0)	0 (0)	0 (0)	0 (0)	0 (0)	0 (0)	0 (0)	0 (0)	0 (0)	1 (100)
Line 11, n (%)	0 (0)	0 (0)	0 (0)	0 (0)	0 (0)	0 (0)	0 (0)	0 (0)	1 (50)	0 (0)
**Treatment duration, months**	—	—	—	29	—	11	—	—	—	3
Median (range)	2.5 (1-10)	7 (1-34)	18.5 (10-25)	—	18 (2-49)	—	1 (0-4)	1 (0-20)	8 (6-10)	—
**Treatment ongoing†, n (%)**	1 (7.1)	3 (18.8)	1 (25)	1 (100)	2 (66.7)	0 (0)	0 (0)	0 (0)	0 (0)	0 (0)

PI3K, Phosphoinositide 3-kinase; PARP, Poly (ADP-ribose) polymerase; CDK, Cyclin-dependent kinase; HER2, Human epidermal growth factor receptor 2; TKI, tyrosine kinase inhibitor; mTOR, mammalian target of rapamycin; FGFR, Fibroblast growth factor receptor; MTB, molecular tumor board; SOC, standard of care; † Last follow-up in July 2024.

Table S4: Outcome of patients treated according to recommendation, grouped by drug class.

**Drug class:**	**PI3K Inhibitor**	**Endocrine therapy**	**PARP Inhibitor**	**CDK4/6 Inhibitor**	**HER2-targeting Antibody**	**HER2-targeting TKI**	**Chemo-therapy**	**Immune Checkpoint Inhibitor**	**mTOR Inhibitor**	**FGFR Inhibitor**
**Total number of treatments**	14	16	4	1	3	1	3	3	2	1
**Treatment stopped prematurely[†], n (%)**	4 (28.6)	1 (6.3)	0 (0)	0 (0)	0 (0)	0 (0)	0 (0)	0 (0)	0 (0)	0 (0)
**Best response[§], n (%)**										
SD/PD <6 months	10 (71.4)	11 (68.8)	0 (0)	0 (0)	1 (33.3)	1 (100)	1 (33.3)	2 (66.7)	0 (0)	0 (0)
SD >6 months	2 (14.3)	4 (25)	2 (50)	0 (0)	0 (0)	0 (0)	0 (0)	1 (33.3)	1 (50)	1 (100)
PR	1 (7.1)	1 (6.3)	1 (25)	1 (100)	1 (33.3)	0 (0)	0 (0)	0 (0)	1 (50)	0 (0)
CR	0 (0)	0 (0)	1 (25)	0 (0)	1 (33.3)	0 (0)	1 (33.3)	0 (0)	0 (0)	0 (0)
Lost to follow-up	1 (7.1)	0 (0)	0 (0)	0 (0)	0 (0)	0 (0)	1 (33.3)	0 (0)	0 (0)	0 (0)
**PFS, months**	—	—	—	29	—	5	—	—	—	1
Median (range)	2.5 (0-10)	4.5 (0-35)	16 (4-21)	—	6 (3-25)	—	1 (0-25)	0 (0-20)	7 (6-8)	—
**No progression at last follow-up[‡], n (%)**	1 (7.1)	3 (18.8)	1 (25)	1 (100)	0 (0)	0 (0)	1 (33.3)	1 (33.3)	0 (0)	0 (0)

SD, stable disease; PD, progressive disease; PR, partial response; CR, complete response; PFS, progression-free survival; † Due to side effects or worsening of general condition. § Response was evaluated according to RECIST 1.1 criteria. ‡ Last follow-up in July 2024.

Table S5: Univariate analysis of clinical benefit associated with clinicopathological or molecular variables.

Variable	Category	No Clinical Benefit (%)	Clinical Benefit (%)	OR (95% CI)†	*p*-value†
**Subtype**	HR+HER2+	2 (66.7%)	1 (33.3%)	—	
	HR+HER2-	16 (55.2%)	13 (44.8%)	—	
	HR-HER2-	2 (66.7%)	1 (33.3%)	—	1
**HR status**	Negative	2 (66.7%)	1 (56.3%)	Ref	
	Positive	18 (33.3%)	14 (43.8%)	1.54 (0.07-98.17)	1
**HER2 status**	Negative	18 (56.3%)	14 (43.8%)	Ref	
	Positive	2 (66.7%)	1 (33.3%)	0.65 (0.01-13.72)	1
**Age group**	<60 years	12 (54.5%)	10 (45.5%)	Ref	
	≥60 years	8 (61.5%)	5 (38.5%)	0.76 (0.14-3.69)	0.7372
**ECOG PS group**	0	10 (50%)	10 (50%)	Ref	
	1-4	10 (66.7%)	5 (33.3%)	0.51 (0.1-2.41)	0.4916
**TMB group**	<10 Mut/Mb	16 (55.2%)	13 (44.8%)	Ref	
	≥10 Mut/Mb	4 (66.7%)	2 (33.3%)	0.62 (0.05-5.18)	0.6804
**LOH group**	<16	20 (66.7%)	10 (33.3%)	Ref	
	≥16	0 (0%)	5 (100%)	∞ (1.45-∞)	**0.00925**

OR, odds ratio; CI, confidence interval; HR, hormone receptor; HER2, human epidermal growth factor receptor 2; Ref, reference; ECOG PS, Eastern Cooperative Oncology Group Performance Status; TMB, tumor mutational burden; LOH, loss of heterozygosity; † For calculation of OR, 95% CI and p-value, the Fisher's exact test was used, with a *p*-value of <0.05 defining statistical significance (in bold).
